# A Machine-Learning Approach to Target Clinical and Biological Features Associated with Sarcopenia: Findings from Northern and Southern Italian Aging Populations

**DOI:** 10.3390/metabo13040565

**Published:** 2023-04-17

**Authors:** Roberta Zupo, Alessia Moroni, Fabio Castellana, Clara Gasparri, Feliciana Catino, Luisa Lampignano, Simone Perna, Maria Lisa Clodoveo, Rodolfo Sardone, Mariangela Rondanelli

**Affiliations:** 1 Department of Interdisciplinary Medicine, University “Aldo Moro”, Piazza Giulio Cesare 11, 70100 Bari, Italy; roberta.zupo@uniba.it (R.Z.); marialisa.clodoveo@uniba.it (M.L.C.); 2 Endocrinology and Nutrition Unit, Azienda di Servizi alla Persona “Istituto Santa Margherita”, University of Pavia, 27100 Pavia, Italy; alessia.moroni02@universitadipavia.it (A.M.); clara.gasparri01@universitadipavia.it (C.G.); 3 Unit of Data Sciences and Technology Innovation for Population Health, National Institute of Gastroenterology IRCCS “Saverio de Bellis”, Research Hospital, Castellana Grotte, 70013 Bari, Italy; luisa.lampignano@irccsdebellis.it; 4 Department of Innovation and Smart City, Municipality of Taranto, 74121 Taranto, Italy; feliciana.catino@comune.taranto.it; 5 Department of Food, Environmental and Nutritional Sciences, Division of Human Nutrition, University of Milan, 20133 Milan, Italy; simone.perna@unimi.it; 6 Local Healthcare Authority of Taranto, 74121 Taranto, Italy; rodolfo.sardone@asl.taranto.it; 7 Department of Public Health, Experimental and Forensic Medicine, University of Pavia, 27100 Pavia, Italy; mariangela.rondanelli@unipv.it; 8 IRCCS Mondino Foundation, 27100 Pavia, Italy

**Keywords:** sarcopenia, nutrition, body composition, machine learning, artificial intelligence, elderly, older adults, aging, Salus in Apulia, Italy

## Abstract

Epidemiological and public health resonance of sarcopenia in late life requires further research to identify better clinical markers useful for seeking proper care strategies in preventive medicine settings. Using a machine-learning approach, a search for clinical and fluid markers most associated with sarcopenia was carried out across older populations from northern and southern Italy. A dataset of adults >65 years of age (*n* = 1971) made up of clinical records and fluid markers from either a clinical-based subset from northern Italy (Pavia) and a population-based subset from southern Italy (Apulia) was employed (*n* = 1312 and *n* = 659, respectively). Body composition data obtained by dual-energy X-ray absorptiometry (DXA) were used for the diagnosis of sarcopenia, given by the presence of either low muscle mass (i.e., an SMI < 7.0 kg/m^2^ for males or <5.5 kg/m^2^ for females) and of low muscle strength (i.e., an HGS < 27 kg for males or <16 kg for females) or low physical performance (i.e., an SPPB ≤ 8), according to the EWGSOP2 panel guidelines. A machine-learning feature-selection approach, the random forest (RF), was used to identify the most predictive features of sarcopenia in the whole dataset, considering every possible interaction among variables and taking into account nonlinear relationships that classical models could not evaluate. Then, a logistic regression was performed for comparative purposes. Leading variables of association to sarcopenia overlapped in the two population subsets and included SMI, HGS, FFM of legs and arms, and sex. Using parametric and nonparametric whole-sample analysis to investigate the clinical variables and biological markers most associated with sarcopenia, we found that albumin, CRP, folate, and age ranked high according to RF selection, while sex, folate, and vitamin D were the most relevant according to logistics. Albumin, CRP, vitamin D, and serum folate should not be neglected in screening for sarcopenia in the aging population. Better preventive medicine settings in geriatrics are urgently needed to lessen the impact of sarcopenia on the general health, quality of life, and medical care delivery of the aging population.

## 1. Introduction

As the burden of population aging increases [1], a multidisciplinary research effort is needed to fill the knowledge gap around risk biopaths and to foster preventive strategies against sarcopenia, a multifactorial syndrome characterized by a progressive and generalized loss of skeletal muscle mass as well as poor physical endurance, which is often combined with subclinical systemic inflammation [2]. Such a decline in skeletal muscle function is a nearly inevitable part of the aging process and has a considerable impact on health care costs and quality of life, since it raises the chance of negative outcomes including falls, fractures, physical impairments, and mortality [3].

Sarcopenia is a geriatric giant triggered by adverse muscle changes commonly experienced late in life. According to the Revised European Consensus on Definition and Diagnosis (EWGSOP2) [4], the dimensions that best define sarcopenia are low levels of three parameters: muscle strength, muscle quantity/quality, and physical performance, of which the latter is an indicator of severity. Hence, poor muscle quantity and quality confirms the presence of sarcopenia, whereas low physical performance clearly rates its severity. Moreover, to detect this condition early in clinical settings, the consensus group focused on a novel algorithm path, so called FACS (Find—Assess—Confirm—Severity), that also took into account the probability of sarcopenia, with reference to the SARC-F questionnaire or the calf circumference. As for the assessment of the muscle mass, a large number of tools are detailed in the literature, but only a few are effectively applicable in the clinical setting and therefore considered in the consensus. Of these, MRI and computed tomography (CT) are the gold standard, but they cannot really be applied in a context beyond research and thus are poorly understood in the clinical setting [5]. Dual-energy X-ray absorptiometry (DXA) is the most available, reliable, and feasible approach to directly assess body composition variables [6], while bioelectrical impedance analysis (BIA) is an alternative indirect method—low-cost compared to DXA—that can be used to screen a much larger population, as DXA is not handheld and cannot be applied in specific populations (e.g., pregnant women, bedridden patients, etc.).

Understanding multimodal indicators that can successfully anticipate the onset of sarcopenia and avert the deleterious cascade of late-life multimorbidity remains an open issue from a preventative standpoint. Given sarcopenia as a multifactorial condition, a single fluid or clinical marker cannot be easily pinpointed or be helpful, and thus focus turns to the implementation of a panel that includes multidomain markers. Further, ideal markers of sarcopenia should be valid, replicable, reliable, specific, affordable, and easily available [7]. In the field of nutrition, the scientific community openly acknowledges haemoglobin, albumin, leptin, uric acid, iron, and vitamin D, amongst others, in predicting the risk of sarcopenia [8,9,10].

Of note, the heterogeneous biological, clinical, and social complexities involving the individual, as well as the different characteristics of the personnel and place where such assessments are conducted, inevitably play a role in decisions about risk variables, their cut-points, and their ranking [11,12]. For these reasons, there could be arguments that a single gold standard variable as well as the best cut-points could translate poorly from the epidemiology field and computational modelling to the real clinical practice. To address this challenge and add to literature surrounding the biopathways of sarcopenia, this research sought to generate scientific evidence by testing a convenience sample composed of two subsets, i.e., one clinical-based and one population-based, both aged over 65 years. Machine-learning-based techniques were implemented and compared in order to evaluate the clinical and fluid markers most associated with the sarcopenia condition across the two population settings.

## 2. Materials and Methods

### 2.1. Study Population

#### 2.1.1. Northern-Italy Population Subset (Santa Margherita Institute, Pavia)

We enrolled male and female subjects aged ≥65 years, with a body mass index (BMI) between 20 and 30 kg/m^2^ [13], who are outpatients at the metabolic rehabilitation unit of the Santa Margherita Institute, Department of Public Health, University of Pavia. Subjects with the following conditions were excluded from the study: severe kidney disease (glomerular filtration rate < 30 mL/min), moderate-to-severe hepatic failure (Child–Pugh Class of B or C), endocrine diseases associated with disorders of calcium metabolism (with the exception of osteoporosis), psychiatric disorders, and cancer (in the previous 5 years). The recruitment period was between January 2020 and January 2022. Informed consent was obtained from all subjects involved in the study. The study was conducted according to the guidelines of the Declaration of Helsinki, and approved by the Institutional Review Board of IRCCS S. De Bellis (protocol code n. 68, 9 April 2019).

#### 2.1.2. Southern-Italy Population Subset (the Salus in Apulia Study)

Participants of the Salus in Apulia population-based study were recruited from the electoral rolls of Castellana Grotte (Bari, Apulia, Southern Italy). The recruiting and evaluation centre was the National Institute of Gastroenterology IRCCS “S. De Bellis” Research Hospital, and the initiative was supported by the Italian Ministry of Health and the Apulia Regional Government. The Salus is an ongoing longitudinal population-based study, activated in 2014, of a representative population of residents in Castellana Grotte (Apulia, southern Italy) who were 65 years of age or older at the time of initial recruitment. While the minimum age of 65 was required for enrolment in the Salus, conversely, the exclusion criteria were lack of mental capacity to express consent, having digestive tract cancers or other malignancies, including dementia and motoneuron diseases, or being under major therapies, which could affect nutritional/physical status. The study design and data collection method are detailed elsewhere [14,15]. Briefly, the entire sampling frame consisted of the 4021 elderly residents in the health registry of the Apulia Region as of 31 December 2014. The study was born as multidisciplinary, including the assessments of the cognitive, sensory, physical, and nutritional domains, as illustrated in some of our previous work [16], and aimed to search for new biological and phenotypic determinants to predict and prevent risky trajectories of aging. Specifically, the data used for the present study came from a subset of the Salus, which included 479 elders who had undergone all examinations required for the purposes of this study. The IRB approved the study of the lead institution, the National Institute of Gastroenterology and Research Hospital “Saverio de Bellis”, and all subjects completed informed consent forms before their evaluation. The study met the principles of the Helsinki Declaration and adhered to the “Standards for Reporting Diagnostic Accuracy Studies” (STARD) guidelines (http://www.stard-statement.org/, accessed on 12 January 2023) and the “Strengthening the Reporting of Observational Studies in Epidemiology” (STROBE) guidelines.

### 2.2. Fluid Biomarker Assessment

A blood sample was collected in the morning after overnight fasting to measure the levels of fasting blood glucose (FBG), glycated haemoglobin (HbA1c), total cholesterol, high-density lipoprotein (HDL) cholesterol, low-density lipoprotein (LDL) cholesterol, and triglycerides, using standard automated enzymatic colorimetric methods (AutoMate 2550, Beckmann Coulter, Brea, CA, USA) under strict quality control. LDL cholesterol was calculated using the Friedewald equation. Plasma glucose was determined using the glucose oxidase method (Sclavus, Siena, Italy). Blood cell count was determined by a Coulter haematology analyser (Beckman–Coulter, Brea, CA, USA). Serum FT3, FT4, and TSH were measured using a competitive photometric method based on the solid-phase antigen-linked technique (LIASON FT3, LIASON FT4, LIASON TSH, Dia-Sorin, Saluggia, Italy). Serum high-sensitivity C-reactive protein (CRP) was assayed using a latex particle-enhanced immunoturbidimetric assay (Kamiya Biomedical Company, Seattle, WA, USA) (reference range: 0–5.5 mg/L; inter-assay coefficient of variation: 4.5%). Serum 25(OH)D was quantified by a chemiluminescence method (Diasorin Inc., Stillwater, MN, USA), and all samples were analysed in duplicate.

### 2.3. Clinical and Physical Assessment

Height was measured to the nearest 0.5 cm using a wall-mounted stadiometer (Seca 711; Seca, Hamburg, Germany). Body weight was determined at the time of DXA to the nearest 0.1 kg using a calibrated balance beam scale (Seca 711; Seca, Hamburg, Germany). BMI was calculated as weight in kilograms divided by height in metres squared (kg/m^2^). Low physical performance was assessed using the Short Physical Performance Battery (SPPB), an objective tool for measuring the physical performance status of the lower extremities [17]. The SPPB is based on three timed tasks: standing balance, walking speed, and chair sit-to-stand tests [12]. The timed results of each subtest were rescaled according to the predefined cutoff points, obtaining a score ranging from 0 (worst performance) to 12 (best performance). A cutoff value of 8 in the SPBB score was considered to indicate low physical performance, in accordance with both EWGSOP panels [4,18]. Handgrip strength (HGS) was assessed using the Jamar Plus Digital Hand Dynamometer (Patterson Medical, Cedarburg, WI, USA). Seated with arms 90 degrees to the sides, 2 trials were taken per arm in an alternating fashion with 30 s of rest between trials. The highest reading was recorded [4].

Bone mineral density (BMD) and whole-body lean mass were measured using DXA (Discovery WI, Hologic, Inc., Marlborough, MA, USA). The skeletal muscle mass index (SMI) was defined as the sum of the muscle masses of the four limbs as appendicular skeletal muscle mass divided by squared height.

Whole-body lean mass (kg) was taken as the sum of the fat-free, bone-free mass of the arms and legs as lean mass. According to the operational definition by the European Working Group on Sarcopenia in Older People (EWGSOP2) [4], the diagnosis of sarcopenia was given by the presence of both low muscle mass (that is, an SMI < 7.0 kg/m^2^ for males or <5.5 kg/m^2^ for females) [4], and low muscle strength, as defined by a low handgrip strength (HGS), that is, an HGS < 27 kg for males or <16 kg for females, or low a physical performance (that is, an SPPB ≤ 8).

### 2.4. Statistical Analysis

The entire sample was first divided according to the population setting, i.e., subjects from the Salus in Apulia population-based study and those from the clinical setting of the Santa Margherita Hospital, in order to assess the overlap of the samples (Table 1). Then, the overall population was further subdivided according to the outcome variable, that is, the sarcopenia condition (presence/absence), and groups were compared to describe the clinical and functional differences in terms of frequency and associations (Table 2).

Normal distributions of quantitative variables were tested using the Kolmogorov–Smirnov test. Data are reported as mean ± standard deviation (M ± SD) for continuous measures and frequency and percentages (%) for all categorical variables. In order to focus on the practical differences between the groups in terms of effect size (ES) [19], differences between continuous variables, between the groups, were calculated using Wilcoxon’s effect size difference between ranks, and their 95% confidence intervals (CI) to assess the magnitude of ES [20]. Prevalence differences were calculated to assess differences between categorical variables.

**Figure 1:**
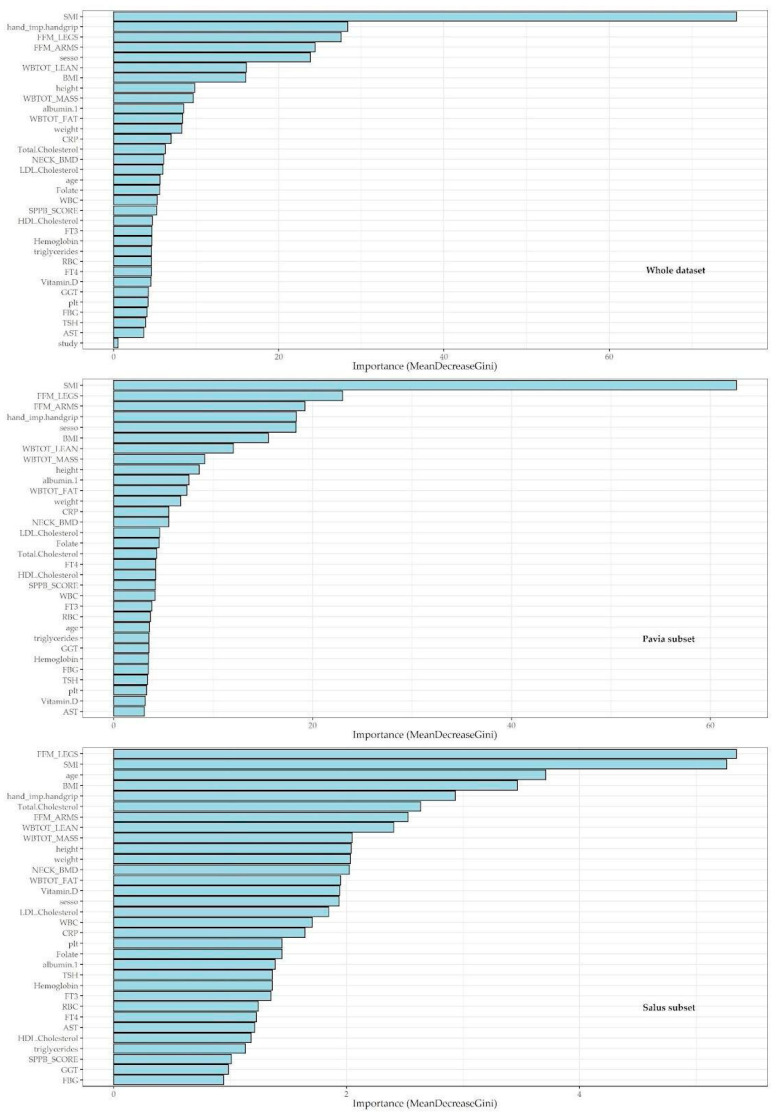
Variable-importance plots for random forest regression models with sarcopenia status as the dependent variable and other collected variables as regressors.

**Figure 2:**
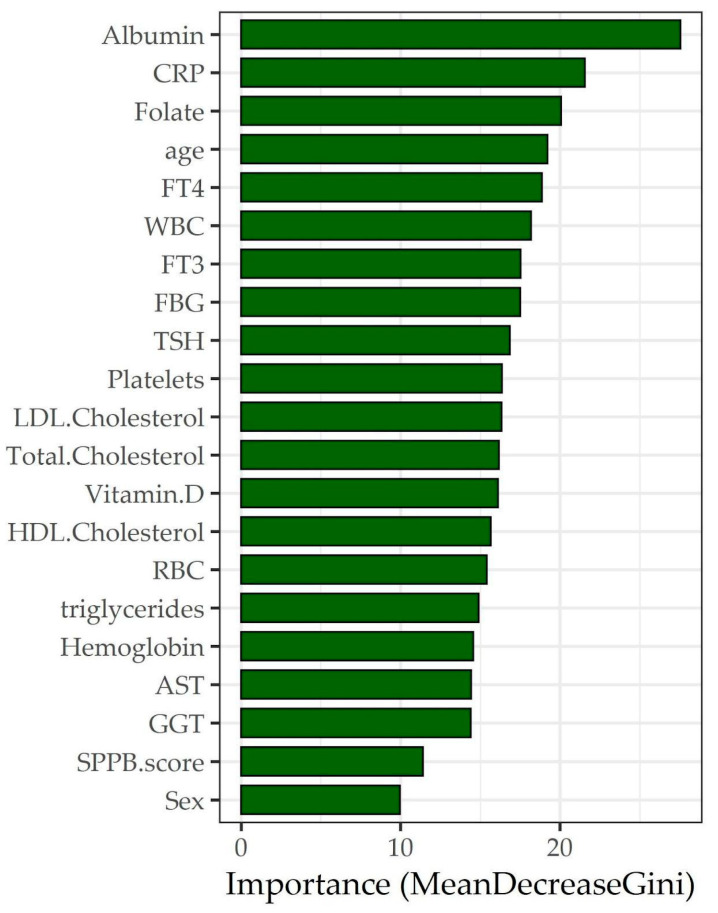
Variable-importance plot for the random forest regression model with sarcopenia status as the dependent variable and socio-demographic and haematochemical variables as regressors.

A machine-learning feature-selection approach, the random forest (RF), was employed to identify the most predictive features in the dataset for the sarcopenic condition considering every possible interaction between them, considering also the non-linear relationships that classical models could not assess (Figure 1). Three RF regression models were built. The first model also included the variables used for the detection of sarcopenia in order to assess which variables were most important in the algorithm of detection using the Mean Decrease Gini. The variables SMI and SPPB were used within an ensemble learning approach in order to highlight possible relationships even in light of variables known to be associated with the condition of sarcopenia. Such an inclusion is therefore useful in order to highlight possible interactions of a nonparametric nature between predictive factors in the classification of sarcopenia. The second RF regression model was performed subdividing according to the clinical study centre in order to assess if there were differences in the association ranking due to the clinical setting. A third RF regression was performed using only socio-demographic and haematochemical variables in order to assess which variables were most associated with the sarcopenia condition using the Mean Decrease Gini (Figure 2).

The accuracy of the logistic regression model was calculated using a first confusion matrix, as shown in Table 3. A logistic regression model was performed on the sarcopenia condition as a dependent variable and sociodemographic and blood chemistry parameters as regressors (Table 4) in order to assess differences between the parametric and non-parametric approach in the prediction of sarcopenia. The accuracy of the third RF regression model was calculated using a further confusion matrix, as shown in Table 5.

## 3. Results

A total of 1791 subjects made up the entire sample; of these, the majority (*n* = 1312, 73.3%) came from the clinical setting of northern Italy. Age over 65 years was a common feature of both samples, and the mean age (±standard deviation, SD) was 79.79 ± 7.18 years and 74.81 ± 5.67 years for the northern and southern Italian populations, respectively.

Table 1 shows a description of the entire sample according to the population setting, i.e., northern (clinical setting) versus southern (population-based setting) Italy. Here, when analysing the between-group practical differences in terms of effect size (ES), as defined by Wilcoxon’s effect size difference and respective 95% confidence intervals (CI), the age (ES: 0.32, 95%CI 0.28–0.36) and sex (ES: 19.10, 95%CI 14.01–24.18) showed significant differences, meaning that the northern Italian sample was older and included more females than the southern counterpart. There was a significantly higher prevalence of sarcopenia in the northern sample (12.6% vs. 7.3%) compared to the southern sample (ES: −5.27, 95%CI −8.21 to −2.33). Along these lines, moving toward DXA-derived body composition variables, significant differences showed up for arm free-fat mass (FFM), whole-body lean mass, and whole-body fat mass. That is, the southern sample showed better values for FFM (ES: 0.20, 95%CI 0.16 to 0.24), whole-body lean mass (ES: 0.20, 95%CI 0.16 to 0.25), and fat mass (ES: 0.34, 95%CI 0.30 to 0.38) than the northern counterpart. Moreover, body weight (ES: 0.33, 95%CI 0.30 to 0.37) and BMI (ES: 0.26, 95%CI 0.22 to 0.30) were also significantly higher in the Apulian sample. Physical function variables were overall better in the population-based southern sample, as indicated by the handgrip strength (ES: 0.24, 95%CI 0.20 to 0.29) and SPPB score (ES: 0.28, 95%CI 0.25 to 0.33). Also, femoral neck BMD followed the trend between the two samples (ES: 0.04, 95%CI 0.01 to 0.09). As for the fluid biomarker pattern, significantly better levels of albumin (ES: 0.37, 95%CI 0.34 to 0.41), folate (ES: 0.11, 95%CI 0.08 to 0.16), vitamin D (ES: 0.54, 95%CI 0.51 to 0.58), haemoglobin (ES: 0.35, 95%CI 0.31 to 0.39), RBC (ES: 0.38, 95%CI 0.34 to 0.42), triglycerides (ES: 0.13, 95%CI 0.09 to 0.18), and HDL cholesterol (ES: 0.12, 95%CI 0.08 to 0.17) emerged for the southern population compared with the northern counterpart.

Following the same analytical approach, Table 2 shows a description of the whole sample by sarcopenia condition (presence/absence). To substantiate the internal validity of the findings, sarcopenic subjects were predominantly older (ES: 0.16, 95%CI 0.12 to 0.21) and male (ES: 24.46, 95%CI 17.20 to 31.72). Our findings indicated significantly lower values of albumin (ES: 0.15, 95%CI 0.12 to 0.22), BMI (ES: 0.33, 95%CI 0.30 to 0.37), FFM of the arms (ES: 0.19, 95%CI 0.15 to 0.25), FFM of the legs (ES: 0.29, 95%CI 0.25 to 0.33), FT3 (ES: 0.13, 95%CI 0.09 to 0.18), handgrip strength (ES: 0.20, 95%CI 0.16 to 0.24), HDL cholesterol (ES: 0.08, 95%CI 0.04 to 0.13), haemoglobin (ES: 0.09, 95%CI 0.05 to 0.14), LDL cholesterol (ES: 0.05, 95%CI 0.01 to 0.10), femoral neck BMD (ES: 0.06, 95%CI 0.02 to 0.11), RBC (ES: 0.07, 95%CI 0.03 to 0.12), SMI (ES: 0.40, 95%CI 0.37 to 0.44), TSH (ES: 0.04, 95%CI 0.01 to 0.08), vitamin D (ES: 0.04, 95%CI 0.01 to 0.09), whole-body fat mass (ES: 0.26, 95%CI 0.23 to 0.31), whole-body lean mass (ES: 0.19, 95%CI 0.15 to 0.24), and body weight (ES: 0.26, 95%CI 0.22 to 0.30) for the sarcopenic population compared with the counterpart. Conversely, the sarcopenic sample showed significantly higher values of CRP (ES: 0.11, 95%CI 0.06 to 0.16) and WBC (ES: 0.10, 95%CI (0.06 to 0.15) than the non-sarcopenic counterpart.

Figure 1 shows a plot of important variables from the RF regression model with sarcopenia condition as the dependent variable and the other variables as regressors. The following rationale guided the selection procedure. The first model was run including both the fluid markers and the functional domains of sarcopenia to evaluate which variables were the most important to be included in the algorithm of sarcopenia detection by using the Mean Decrease Gini. The graph showed that SMI (Mean Decrease Gini greater than 60), followed by handgrip strength, FFM (arms and legs), sex (all showing a 20 to 40 Mean Decrease Gini), BMI, and total lean body mass (Mean Decrease Gini over 15) were the most relevant. The second RF regression model was run separately after dividing the sample into two subsets according to the population setting (northern vs. southern Italy, i.e., clinical vs. population-based setting) to assess whether there were differences in the importance ranking due to the different settings. Here, the ranking showed almost overlapping findings, that is, SMI, handgrip strength, FFM (legs and arms), and sex being the top variables of importance in both subsets. A third RF regression was performed using only the socio-demographic and haematochemical variables to assess which variables were most associated with the condition of sarcopenia in the total sample. Here, albumin, CRP, and folate were shown to be top-ranked (Mean Decrease Gini: 27.5, 21.56, and 20.08, respectively) (Figure 2). The reliability of the third RF regression model was evaluated using a confusion matrix (Table 3). The findings showed an accuracy of 94.57 (95%CI 92.15 to 96.42), a sensitivity of 1.00, and a specificity of 0.94.

To explore the differences between the parametric and nonparametric approaches in predicting sarcopenia, a logistic regression model was run on sarcopenia condition as the dependent variable and sociodemographic and fluid biomarkers variables as regressors (Table 4). Male gender showed the strongest association with sarcopenia (OR: 4.384, 95%CI 3.027 to 6.351). Slightly at risk were those subjects showing lower SPPB scores (OR: 0.906, 95%CI 0.847 to 0.969), serum folate (OR: 1.022, 95%CI 1.005 to 1.038), and vitamin D (OR: 1.015, 95%CI 1.002 to 1.028). Again, the reliability of the logistic regression model was evaluated using a confounding matrix (Table 5). The findings showed an accuracy of 89.89 (95%CI 87.70 to 90.62), a sensitivity of 14.50, and a specificity of 99.37.

## 4. Discussion

This study aimed to investigate clinical and fluid markers most associated with the condition of sarcopenia by implementing a machine-learning-based approach across two subsets (clinical and population-based) of adults over 65 years of age to provide evidence on how to better set up preventive strategies in sarcopenia settings. The research was carried out involving populations from northern and southern Italy, respectively from the Santa Margherita Clinic (Pavia, northern Italy) and the Salus in Apulia population-based study (Apulia, southern Italy). The top-ranked association variables for sarcopenia in RF selection overlapped across the two population subsets, and included SMI, HGS, FFM of legs and arms, and sex. Given these similarities in terms of clinical features, we implemented parametric and nonparametric analysis on the whole sample to investigate those variables most associated with sarcopenia, and found albumin, CRP, folate, and age to rank high in RF selection, while sex, folate, and vitamin D were most relevant in logistic.

First, to substantiate the internal validity of our findings, it is worth noting that statistical analyses by sarcopenia condition (presence/absence) showed a higher proportion of males as well as older age, and lower levels of albumin, haemoglobin, vitamin D, and BMI in the sarcopenic population. In terms of comparison, our clustered analyses by population setting showed meaningful differences between subsets in the prevalence of sarcopenia (higher in the clinical setting population, i.e., 12% compared to 7% of the population-based counterpart) and, in turn, fluid biological markers and functional proxies. The clinical setting population showed lower (worst) values in FFM legs and arms, HGS, SPPB, and BMI, as well as lower serum levels of albumin, folate, vitamin D, haemoglobin, RBC, triglycerides, and HDL cholesterol. Functional dimensions such as HGS and SPPB scoring were also worse in the clinical subset. These data fit well within a geriatric outpatient/clinical healthcare setting, as the latter assumes that subjects come to the hospital to fix some medical issue, whereas in a population-based setting, subjects are usually recruited to participate in data collection for research purposes because they fit the purposes of the study well, and thus come to the hospital for a visit without a specific health concern to fix. In our specific case, subjects from the north were recruited in a clinical setting that included both outpatients and inpatients, and were therefore expected to be more frail and physically impaired than the general population recruited in the southern sample. This view most likely explains the poorer general health status of the clinical subset. Moreover, the physiological path of reduction in testosterone, the hormone that drives protein synthesis and muscle development, may then elucidate the higher male incidence of sarcopenia already acknowledged by the scientific community [21]. Basically, testosterone works as the fuel that powers muscle building.

The RF plot of important variables for sarcopenia algorithm showed SMI, HGS, FFM (arms and legs), sex, and BMI as the most relevant. Due to the predominance of skeletal muscle in the arms and legs, is not surprising that both lean soft tissues are key and are actually embedded in the SMI as muscle proxies in the consensus panel. For BMI, the role in relation to sarcopenia can be explained in both excess and deficit values. On the one hand, weight gain and obesity can speed up the onset and the progression of sarcopenia directly or indirectly. For example, in fat tissue of obesity phenotypes [22], the accumulation of pro-inflammatory macrophages and other immune cells, as well as the dysregulated production of various adipokines together with cytokines released by immune cells, create a prolonged local pro-inflammatory state [23,24]. In addition, over-production and impaired lipid storage capacity is a feature of adipose tissue in obesity, which accumulates ectopically in skeletal muscle. These intramuscular lipids and their products can result in mitochondrial dysfunction and increased secretion of certain pro-inflammatory myokines with the potential to induce muscle dysfunction. On the other hand, reduced BMI may be equally troublesome as it would mean a reduced fat mass, that is believed to be an energy reserve in older people and helps them survive disease and chronic conditions [25]. It has also been hypothesized that individuals with higher fat mass may have higher protein intake, which is a protective factor against sarcopenia [26]. Therefore, maintaining a healthy weight is important for older adults to preserve muscle mass and strength.

In a further RF plot of significance, we found overlapping top-ranked association variables across the two population settings (i.e., SMI, HGS, FFM of legs and arms, and sex) showing how the clinical and population-based subsets were not much divergent. Therefore, an additional RF plot was built without splitting the two samples to get a selection of variables most associated with sarcopenia, and found that albumin, CRP, and folate ranked high. A further comparative logistic analysis found sex, folate, and vitamin D among the most closely associated, although the latter lacked significance. These findings are discussed below.

It is well-acknowledged that serum albumin concentration may be an indicator of individual nutritional status [27,28], with lower values indicating a decrease in protein reserve, stimulating catabolic processes that lead to muscle breakdown [29]. There is also a body of research indicating the antioxidant properties of albumin, showing that albumin is a specific modulator of cellular glutathione, one of the body’s major antioxidants [30]. Oxidative damage may play a crucial role in skeletal muscle decline with aging. Furthermore, increased concentrations of free cortisol have been observed in hypoalbuminemic individuals, and this other biological pathway potentially stimulates muscle breakdown, especially in inactive people. Albumin has also been shown to activate the phosphatidyl-inositol 3-kinase pathway, thus mediating muscle breakdown.

For serum folate, our findings are in line with other studies revealing serum folate levels significantly correlated with reduced lower limb strength and grip in the elderly over 65, especially in women [31]. Findings from other research suggest that folate deficiency is associated with a decline in muscle strength, and that a reduced dietary micronutrient intake [32,33], including folate, has an important impact on muscle health, as indicated by decreased ability to generate strength and endurance as well as reduced physical activity. We therefore suggest that the effect of folate deficiency on measures of strength and physical performance involves biological mechanisms that could include specific folate activities in addition to the homocysteine pathway, such as neurotransmitter synthesis, myelination, DNA and protein synthesis, DNA methylation, and epigenetic regulation [34].

For vitamin D, epidemiological studies showed clinical correlations between vitamin D deficiency and sarcopenia, such that this lipoprotein is attracting more and more attention among the scientific community. In the general population, low serum vitamin D concentration has been significantly found to be associated with a higher prevalence of sarcopenia and loss of physical performance, such as walking speed [35,36]. Several meta-analyses and RCT studies have also demonstrated the positive effects of vitamin D supplementation, such as improved overall muscle strength (particularly of lower limb muscles) and decreased standing time [37,38,39].

Last, regarding the inflammatory pathway, it has long been hypothesized that higher levels of inflammatory markers play a role in functional decline in aging populations [40,41]. Cross-sectional and longitudinal studies consistently demonstrated associations between high levels of interleukins, especially interleukin-6 (IL-6) [42], or CRP and poor physical performance and disability. The causal pathway running from inflammation to disability is suggested to involve catabolic effects of inflammatory markers on muscles. To further substantiate our findings, there is evidence that in older men and women, high levels of CRP are associated with a 2- to 3-fold increased risk of losing more than 40% of muscle strength over 3 years [43].

When comparing sarcopenia to other studies, we can look at what has been found in other reports. For example, among elderly people aged ≥ 65 years admitted to daycare centres in Taiwan [44], calf circumference, Mini Nutritional Assessment, dementia, and BMI were factors associated with sarcopenia, diagnosed, however, using Asian Working Group for Sarcopenia (AWGS) criteria. In another report using data from elderly people in Brazil and the EWGSOP criteria for sarcopenia diagnosis [45], older age, cognitive impairment, lower income, smoking, and undernutrition and undernutrition risk were factors associated with sarcopenia. In a report that used machine learning like ours as a data analysis approach [46], the top risk factors in men were BMI, red blood cell count (RBC), blood urea nitrogen (BUN), vitamin D, ferritin, fibre intake (g/d), primary diastolic blood pressure, white blood cell count (WBC), fat intake (g/d), age, glutamic–pyruvic transaminase, niacin intake (mg/d), protein intake (g/d), fasting blood glucose, and water intake (g/d). Inevitably, the most important risk factors in women were BMI, water intake (g/d), WBC, RBC count, iron intake (mg/d), BUN, high-density lipoprotein, protein intake (g/d), fibre consumption (g/d), vitamin C (mg/d), parathyroid hormone, niacin intake (mg/d), carotene intake (μg/d), potassium intake (mg/d), calcium intake (mg/d), sodium intake (mg/d), retinol intake (μg/d), and age. The population setting, study setting, exposome, and diagnostic criteria used for diagnosis certainly create heterogeneity in the findings, and making it unfeasible to draw consistent conclusions.

### Strengths and Limitations

The strengths of this study include the large sample size, the multiple anthropometric and clinico-metabolic variables collected and correlated with sarcopenia, the use of DXA as the gold standard for body composition and thus for reliability of findings, and the employment of robust statistical algorithms and evidence-based references. Further, double recruitment and data collection on two different populations lacks any previous research to compare to in Italy. However, some limitations should be acknowledged. The cross-sectional nature precludes causal inference on outcomes, and although comprehensive, the database lacked a broader assessment of fluid markers. Moreover, by skimming the dataset for variables shared by the two population samples, a more comprehensive assessment of the biological markers panel was not allowed.

## 5. Conclusions

Albumin, CRP, vitamin D, and serum folate should not be overlooked when screening for sarcopenia in geriatric settings, particularly in the male population. Improving the health burden and quality of life of the aging population is urgently needed. Employing multidimensional methodology to model risk management pathways may provide a way to stratify the risk of sarcopenia in preventive medicine settings, and thus ease the identification of a deteriorating health status in the aged population.

## Figures and Tables

**Table 1:** Description of the whole sample according to population setting, i.e., northern (clinical setting) versus southern (population-based setting) Italy.

	Clinical-Based Subset (Pavia)		Population-Based Subset (Apulia)		
	Mean ± SD	Median (IQR)	Mean ± SD	Median (IQR)	Wilcoxon’s ES
Prop. (%)	1312 (73.30)		479 (26.70)		
Age (years)	79.79 ± 7.18	80 (10)	74.81 ± 5.67	74.09 (7.58)	**0.32 (0.28 to 0.36)**
Sex					
Female	949 (72.30)		255 (53.20)		**19.10 (14.01 to 24.18)**
Male	363 (27.70)		224 (46.80)		
Sarcopenia	165 (12.60)		35 (7.30)		**−5.27 (−8.21 to −2.33)**
Albumin (g/dL)	3.67 ± 0.51	3.74 (0.67)	4.08 ± 0.42	4.05 (0.4)	**0.37 (0.34 to 0.41)**
AST (U/L)	18.65 ± 17.7	14 (10)	20.39 ± 9.33	19 (8)	**0.26 (0.22 to 0.30)**
BMI (kg/m^2^)	25.57 ± 5.88	24.7 (6.7)	29.75 ± 4.94	29.32 (6.08)	**0.26 (0.22 to 0.30)**
CRP (mg/dL)	1.25 ± 2.63	0.35 (0.84)	0.43 ± 0.62	0.43 (0.33)	0.02 (−0.01 to 0.05)
FBG (mg/dL)	108.19 ± 41.06	96 (33)	102.68 ± 25.31	96 (21)	0.01 (−0.03 to 0.03)
FFM arms (kg)	4.12 ± 1.38	3.86 (1.70)	4.71 ± 1.354	4.51 (2.15)	**0.20 (0.16 to 0.24)**
FFM legs (kg)	13.419 ± 3.21	12.98 (4.33)	13.24 ± 3.02	13.14 (4.71)	0.02 (−0.03 to 0.04)
Folate (ng/mL)	9.13 ± 9.19	5.9 (7.82)	8.98 ± 5.95	7.6 (5.3)	**0.11 (0.08 to 0.16)**
FT3 (pmol/L)	2.32 ± 0.53	2.3 (0.64)	3.32 ± 0.39	3.32 (0.46)	**0.67 (0.65 to 0.70)**
FT4 (pmol/L)	5.76 ± 5.74	1.46 (9.78)	0.97 ± 0.66	0.9 (0.19)	**0.54 (0.51 to 0.58)**
GGT (U/L)	32.98 ± 42.09	20 (18)	19.44 ± 16.61	15 (9)	**0.21 (0.17 to 0.26)**
HGS (kg)	18.68 ± 7.74	18 (9.33)	23.13 ± 8.28	22 (11.67)	**0.24 (0.20 to 0.29)**
HDL Cholesterol (mg/dL)	47.96 ± 17.65	47 (21)	52.41 ± 14.02	52 (17)	**0.12 (0.08 to 0.17)**
Height (cm)	156.79 ± 9.62	155 (13)	157.45 ± 9.03	157 (13)	0.04 (−0.01 to 0.09)
Haemoglobin (g/dL)	12.35 ± 1.72	12.4 (2.2)	13.68 ± 1.44	13.7 (1.8)	**0.35 (0.31 to 0.39)**
LDL Cholesterol (mg/dL)	111.76 ± 37.32	107.9 (48.85)	104.9 ± 32.66	104 (49)	**0.07 (0.20 to 0.12)**
Neck BMD	0.74 ± 0.18	0.73 (0.2)	0.77 ± 0.58	0.7 (0.17)	**0.04 (0.01 to 0.09)**
Platelets (103 cells/mm^3^)	247.02 ± 103.99	238.6 (103.72)	230.55 ± 71.2	226 (75.5)	**0.07 (0.03 to 0.12)**
RBC (106 cells/mm^3^)	4.18 ± 0.65	4.17 (0.69)	4.66 ± 0.55	4.66 (0.62)	**0.38 (0.34 to 0.42)**
SMI (kg/m^2^)	7.08 ± 1.37	7 (1.72)	7.17 ± 1.18	7.1 (1.75)	0.03 (−0.01 to 0.08)
SPPB score	6.26 ± 3.04	6 (5)	8.27 ± 2.86	9 (5)	**0.28 (0.25 to 0.33)**
Total Cholesterol (mg/dL)	185.68 ± 41.34	184.5 (58)	180.14 ± 37.31	179 (50.5)	**0.05 (0.01 to 0.10)**
Triglycerides (mg/dL)	132.2 ± 80.08	111 (75.25)	113.56 ± 75.43	93 (63.5)	**0.13 (0.09 to 0.18)**
TSH (µU/mL)	2.13 ± 2.28	1.5 (1.61)	1.87 ± 1.76	1.55 (1.29)	0.01 (−0.03 to 0.03)
Vitamin D (nmol/L)	13.29 ± 10.77	10.2 (10.2)	27.98 ± 12.69	26.4 (12.7)	**0.54 (0.51 to 0.58)**
WBC (103 cells/mm^3^)	7.01 ± 3.14	6.54 (2.41)	6.36 ± 1.81	6.09 (2.13)	**0.10 (0.06 to 0.15)**
Whole Body Fat (kg)	22.60 ± 10.46	21.30 (13.26)	30.04 ± 8.943	28.68 (11.84)	**0.34 (0.30 to 0.38)**
Whole Body Lean Mass (kg)	38.84 ± 7.928	37.29 (10.52)	42.77 ± 8.503	41.61 (13.56)	**0.20 (0.16 to 0.25)**
Whole Body Mass (kg)	63.43 ± 15.59	61.70 (20.01)	72.82 ± 13.93	72.34 (18.93)	**0.28 (0.25 to 0.33)**
Weight (kg)	62.85 ± 15.53	60.55 (19.2)	73.86 ± 14.31	72.6 (18.7)	**0.33 (0.30 to 0.37)**

*n* = 1791. All data are shown as mean ± SD, median (min to max) for continuous variables and as *n* (%) for proportions. Statistically significant data are indicated in bold type. Abbreviations: AST: aspartate aminotransferase; BMI: body mass index; CRP: C-reactive protein; FBG: fasting blood glucose; FFM: fat-free mass; GGT: gamma-glutamyl transferase; HGS: handgrip strength; BMD: bone mineral density; RBC: red blood cells; SMI: skeletal muscle index; SPPB: Short Physical Performance Battery; TSH: thyroid-stimulating hormone.

**Table 2:** Description of the whole sample by sarcopenia condition (presence/absence).

	Without Sarcopenia		With Sarcopenia		
	Mean ± SD	Median (IQR)	Mean ± SD	Median (IQR)	Wilcoxon’s Effect Size
Prop. (%)	1591 (88.80)		200 (11.20)		
Age (years)	78.04 ± 7.1	78 (10.67)	81.79 ± 6.76	82.1 (10)	**0.16 (0.12 to 0.21)**
Sex					
Female	1113 (70.00)		91 (45.50)		**24.46 (17.20 to 31.72)**
Male	478 (30.00)		109 (54.50)		
Population setting					
Pavia subset	1147 (72.10)		165 (82.50)		−10.41 (−16.12 to −4.70)
Apulia subset	444 (27.90)		35 (17.50)		
Albumin (g/dL)	3.81 ± 0.5	3.87 (0.57)	3.53 ± 0.62	3.56 (0.9)	**0.15 (0.12 to 0.22)**
AST (U/L)	18.98 ± 15.17	16 (11)	20.15 ± 20.96	14 (11)	0.03 (−0.01 to 0.08)
BMI (kg/m^2^)	27.35 ± 5.83	26.64 (7)	21.47 ± 3.92	21.3 (5.12)	**0.33 (0.30 to 0.37)**
CRP (mg/dL)	0.9 ± 2.02	0.34 (0.44)	2.1 ± 3.76	0.5 (1.92)	**0.11 (0.06 to 0.16)**
FBG (mg/dL)	106.47 ± 36.6	96 (29)	108.74 ± 44.6	93.5 (35)	0.02 (−0.02 to 0.05)
FFM arms (kg)	4.38 ± 1.38	4.05 (1.91)	3.45 ± 1.24	3.292 (2.00)	**0.19 (0.15 to 0.25)**
FFM legs (kg)	13.70 ± 3.08	13.31 (4.45)	10.73 ± 2.45	10.52 (3.53)	**0.29 (0.25 to 0.33)**
Folate (ng/mL)	8.86 ± 7.9	6.4 (6.8)	10.91 ± 11.75	6.85 (10.85)	0.03 (−0.01 to 0.08)
FT3 (pmol/L)	2.62 ± 0.66	2.55 (0.89)	2.32 ± 0.7	2.32 (0.74)	**0.13 (0.09 to 0.18)**
FT4 (pmol/L)	4.64 ± 5.42	1.21 (9.27)	3.14 ± 4.61	1.18 (0.52)	0.01 (−0.02 to 0.04)
GGT (U/L)	28.72 ± 35.73	18 (15)	34.5 ± 49.29	21 (17)	0.04 (−0.01 to 0.09)
HGS (kg)	20.45 ± 8.19	19 (10.17)	15.26 ± 5.92	14 (9)	**0.20 (0.16 to 0.24)**
HDL Cholesterol (mg/dL)	49.66 ± 16.71	49 (21)	45.11 ± 17.6	44 (21)	**0.08 (0.04 to 0.13)**
Height (cm)	156.79 ± 9.38	156 (13)	158.33 ± 10.05	158 (15)	0.04 (−0.01 to 0.08)
Haemoglobin (g/dL)	12.76 ± 1.74	12.9 (2.3)	12.28 ± 1.8	12.3 (2.35)	**0.09 (0.05 to 0.14)**
LDL Cholesterol (mg/dL)	110.64 ± 36.16	107.4 (48.3)	104.2 ± 36.59	100.2 (52.5)	**0.05 (0.01 to 0.10)**
Neck BMD	0.76 ± 0.35	0.72 (0.19)	0.71 ± 0.22	0.69 (0.24)	**0.06 (0.02 to 0.11)**
Platelets (103 cells/mm^3^)	242.83 ± 95.19	237 (91.45)	240.9 ± 107.23	225.9 (103.33)	0.01 (−0.03 to 0.04)
RBC (106 cells/mm^3^)	4.32 ± 0.66	4.32 (0.75)	4.17 ± 0.63	4.18 (0.73)	**0.07 (0.03 to 0.12)**
SMI (kg/m^2^)	7.29 ± 1.24	7.19 (1.73)	5.59 ± 0.86	5.44 (1.32)	**0.40 (0.37 to 0.44)**
SPPB score	6.94 ± 3.1	7 (4)	5.62 ± 3.03	6 (5)	**0.13 (0.09 to 0.18)**
Total Cholesterol (mg/dL)	185.45 ± 39.86	184 (55)	174.24 ± 43.02	169 (64)	**0.09 (0.05 to 0.14)**
Triglycerides (mg/dL)	127.36 ± 79.02	106 (74)	126.07 ± 81.52	100.5 (63)	0.13 (−0.03 to 0.04)
TSH (µU/mL)	2.1 ± 2.23	1.54 (1.55)	1.74 ± 1.37	1.42 (1.48)	**0.04 (0.01 to 0.08)**
Vitamin D (nmol/L)	17.36 ± 13.01	13.9 (16.85)	16.13 ± 13.31	10.9 (12.43)	**0.04 (0.01 to 0.09)**
WBC (103 cells/mm^3^)	6.76 ± 2.9	6.36 (2.3)	7.46 ± 2.41	6.98 (2.83)	**0.10 (0.06 to 0.15)**
Whole Body Fat (kg)	25.55 ± 10.50	24.49 (14.07)	16.94 ± 7.91	16.58 (10.77)	**0.26 (0.23 to 0.31)**
Whole Body Lean Mass (kg)	40.47 ± 82.44	38.75 (12.35)	35.28 ± 6.91	34.64 (11.30)	**0.19 (0.15 to 0.24)**
Whole Body Mass (kg)	67.47 ± 15.46	66.28 (20.93)	53.78 ± 12.05	53.64 (16.62)	**0.27 (0.24 to 0.32)**
Weight (kg)	67.25 ± 15.83	65.4 (21)	54.23 ± 11.87	54.3 (17.23)	**0.26 (0.22 to 0.30)**

*n* = 1791. All data are shown as mean ± SD, median (min to max) for continuous variables and as *n* (%) for proportions. Statistically significant data are indicated in bold type. Abbreviations: AST: aspartate aminotransferase; BMI: body mass index; CRP: C-reactive protein; FBG: fasting blood glucose; FFM: fat-free mass; GGT: gamma-glutamyl transferase; HGS: handgrip strength; BMD: bone mineral density; RBC: red blood cells; SMI: skeletal muscle index; SPPB: Short Physical Performance Battery; TSH: thyroid-stimulating hormone.

**Table 3:** Confusion matrix (Salus dataset as test dataset) on sarcopenia status.

		Prediction	
		Without	With
Test dataset (Salus)	Without	444 (94.50)	--
	With	26 (5.50)	9 (100.00)
**Accuracy (CI 95%)**			
94.57 (92.15 to 96.42)			
**Sensitivity**			
1.00			
**Specificity**			
0.94			

**Table 4:** Logistic regression model with sarcopenic condition as the dependent variable and all haematochemical features as regressors.

	OR	Stand. Err.	CI 95%	*p*-Value
**(Intercept)**	**0.013**	**1.61**	**0.001 to 0.301**	**<0.01**
**Age (years)**	**1.054**	**0.01**	**1.024 to 1.085**	**<0.01**
**Sex (Male)**	**4.384**	**0.18**	**3.027 to 6.351**	**<0.01**
**SPPB score**	**0.906**	**0.03**	**0.847 to 0.969**	**<0.01**
WBC (103 cells/mm^3^)	1.051	0.01	0.998 to 1.108	0.07
RBC (106 cells/mm^3^)	1.200	0.16	0.860 to 1.674	0.28
Platelets (103 cells/mm^3^)	1.001	0.01	0.999 to 1.002	0.76
FBG (mg/dL)	0.999	0.01	0.995 to 1.003	0.57
Triglycerides (mg/dL)	1.001	0.01	0.989 to 1.013	0.88
Total Cholesterol (mg/dL)	1.004	0.01	0.950 to 1.062	0.87
HDL Cholesterol (mg/dL)	1.001	0.01	0.943 to 1.062	0.98
LDL Cholesterol (mg/dL)	0.997	0.01	0.943 to 1.053	0.90
**TSH (µU/mL)**	**0.871**	**0.01**	**0.784 to 0.969**	**0.01**
**FT3 (pmol/L)**	**0.555**	**0.17**	**0.393 to 0.783**	**<0.01**
**FT4 (pmol/L)**	**0.921**	**0.01**	**0.887 to 0.956**	**<0.01**
CRP (mg/dL)	1.065	0.01	0.999 to 1.135	0.06
**Folate (ng/mL)**	**1.022**	**0.01**	**1.005 to 1.038**	**<0.01**
**Vitamin D (nmol/L)**	**1.015**	**0.01**	**1.002 to 1.028**	**0.01**
Haemoglobin (g/dL)	1.007	0.01	0.873 to 1.16	0.92
GGT (U/L)	0.999	0.01	0.995 to 1.004	0.73
AST (U/L)	0.999	0.01	0.989 to 1.011	0.99
**Albumin (g/dL)**	**0.562**	**0.19**	**0.386 to 0.818**	**<0.01**

Abbreviations: AST: aspartate aminotransferase; BMI: body mass index; CRP: C-reactive protein; FBG: fasting blood glucose; FFM: fat-free mass; GGT: gamma-glutamyl transferase; HGS: handgrip strength; RBC: red blood cells; SMI: skeletal muscle index; SPPB: Short Physical Performance Battery; TSH: thyroid-stimulating hormone.

**Table 5:** Logistic regression model confusion matrix.

		Reference	
		No	Yes
**Prediction**	No	1581	171
	Yes	10	29
**Accuracy**	89.89		
**Sensitivity**	14.50		
**Specificity**	99.37		

## Data Availability

The data presented in this study are available on request from the corresponding author Fabio Castellana. The data are not publicly available due to privacy reasons.
